#  Blood Concentrations of Cadmium and Lead in Multiple Sclerosis Patients from Iran

**Published:** 2016

**Authors:** Mehdi Aliomrani, Mohammad Ali Sahraian, Hamid Shirkhanloo, Mohammad Sharifzadeh, Mohammad Reza Khoshayand, Mohammad Hossein Ghahremani

**Affiliations:** a *Department of Pharmacology and Toxicology, Faculty of Pharmacy, Tehran University of Medical Sciences.*; b *MS Research Center, Department of Neurology, school of Medicine, Tehran University of Medical Sciences.*; c *Iranian Petroleum Industry Health Research Institute (IPIHRI), Tehran, Iran.*; d *Department of Drug & food control, Faculty of Pharmacy, Tehran University of Medical Sciences, Tehran, Iran.*

**Keywords:** Multiple sclerosis, Lead, Heavy metal, Cadmium, Oxidative stress, Disability

## Abstract

Since industrial revolution heavy metals such as lead (Pb) and cadmium (Cd) have been extensively dispersed in environment which, unknown biological effects and prolong biological half-life make them as a major hazard to human health. In addition, the sharp increase in Multiple sclerosis incidence rateshas been recorded in Iran. The propose of this study was to measuring blood lead and cadmium concentration and their correlation with smoking habit in a group of 69 RRMS patients and 74 age/gender-matched healthy individuals resident in Tehran as most polluted city in Iran. All subjects were interviewed regarding age, medical history, possible chemical exposure, acute or chronic diseases, smoking and dietary habits. Blood Pb and Cd levels were measured by double beam GBC plus 932 atomic absorption spectrometer. Our result indicated a significant difference in Cd level (p = 0.006) in which, MS patients had higher blood concentration (1.82 ± 0.13 μg/L) in comparison with healthy individuals (1.47 ± 0.11 μg/L). A comparable blood Cd level to similar recent study (1.78 µg/L vs.1.82 µg/L) was observed. With respect to Pb there was no significant difference between cases and controls, however the geometric means of blood Pb concentration were considerably higher in males than in females in MS patients (57.1 ± 33.7 μg/L *vs*. 36.7 ± 21.9 μg/L. P = 0.02). Taking into consideration tobacco smoking, an elevated contents of each metal were observed in smoker subjects (p<0.0001). A significant correlation between cigarette smoking and risk of multiple sclerosis was shown before. Thus, high level of Cd in smokers might affect the susceptibility to multiple sclerosis and could increase the risk of disease development.

## Introduction

Monitoring the human tissue toxic metals level has been continued for the last couple of decades in developed countries. However, in our country an ordinary range of toxic metals level in human tissues has been rarely measured in recent years. Tehran is the capital of Iran with a population around 14 million where, rapid industrial and economical developments have been resulted in an increase of environmental pollution (1). Among various environmental pollutions in the urban area, heavy metals have the most harmful effect on public health, because they remain in the ecosystem and are not biodegradable (2).

Since the industrial revolution lead and cadmium are extensively dispersed in the environment (3). Moreover, high toxic features of these metals, with long time persistency in the human body (20-35 years) make them as a major hazard to human health (4, 5). It has been observed that lead and cadmium toxicity contributes to a vast variety of important disease conditions such as neurological disorders, cancer, cognitive impairments, hypertension, heart disease and diabetes(6).

Because of unusual physical and chemical characteristics, lead is used in the diverse industrial process (5). The most frequent routes of exposure are soil, products containing lead (paints, gasoline, insecticides, cosmetics, plastics, batteries) and water contaminated by lead pipes. Children gastrointestinal absorption of lead in oral exposure is about 4 times higher than adults (5). Pb binds to hemoglobin in RBCs and gradually released to soft tissues including liver, kidneys, brain and other organs. Accumulation of lead in bones increases its biological half-life to 27 years in the human body (7). Redox-active metals such as lead generate free radical species by participating in the transfer of electrons. The molecular mechanism of lead toxicity is multifactorial as it generates free radical species, decreases glutathione antioxidant sulphydryl pools; inhibits enzyme activity and blocks important trace element absorption (8).

In addition, cadmium is one of the most important toxic metals while the main routes of its exposure are polluted air inhalation, cigarette smoking and foods (9). After body entrance cadmium mainly bond to metallothionein and makes Cd-MT complex. Because of no specific excretion mechanism, it is accumulated to a great extent in kidneys cortex, lungs, pancreas and liver for 20-35 years (10).The precise molecular mechanisms of Cd toxicity are not known however, it has been suggested that Cd indirectly enhances the free radical generation and participates in oxidative stress via Fenton reaction (5).

On the other hand, it has been reported that sharp increase in Multiple sclerosis (MS)incidence rate has been observed in Iran (11). MS is a chronic immune-mediated, inflammatory neurological disease of the central nervous system which attacks the myelinated axons and destroying them in variable degrees (12, 13). Focal inflammatory plaques and axonal loss are considered as a main pathological feature of MS though the question about etiology of MS is still unresolved (14, 15). According to the Multiple Sclerosis international federation, roughly 2.3 million people are affected by MS across the world (16). At the time of diagnosis, about 85% of cases have relapsing–remitting (RRMS) form of MS (17). RRMS is defined by short-term exacerbations of neurologic deficits followed by remission, when symptoms improve or completely disappears (12). Growing evidence suggests that the interplay between genetic pattern and environmental exposure may result in the activation of immune system and neuronal injury. While recent studies have identified that MS disease duration was impacted by some of these environmental risk factors exposure (14). 

In recent years, toxicological researches are significantly focused on the determination of factors influencing heavy metal poisonings and their impact on different medical conditions. So, the objective of this study is to measure blood lead and cadmium concentration in relapsing-remitting multiple sclerosis patients.

## Methods

*Subjects*

To increase sampling reliability, all subjects were selected in the central part of Tehran. Thus, the blood analytical measurements are not affected by subjects area of residence. Following ethics committee approval (by the Institutional Review Board of Medical Ethics, Pharmaceutical Sciences Research Center/Tehran University of Medical Sciences), totally 143 volunteers with moderate socioeconomic position and ordinary dietary habits were registered. Patients group comprised 69 unrelated RR-MS patients with clinically defined disease according to the revised McDonald criteria (18) while control group included 74 healthy volunteers living in the same urban area. All subjects selected by applying exclusion criteria including serious kidney, cardiological, respiratory or liver disease, vegetarian diet, artificial metallic bodies, lithium and thyroid hormone therapy, daily supplement intake and other neurological diseases. The participants were recruited between Sep 2013 and Sep 2014 from the multiple sclerosis department of the Sina hospital, one of the principal teaching hospitals of Tehran University of Medical Sciences. All patients were evaluated during a stable period of their illness and had not received steroid therapy for last 3 months ago. Written permission was obtained from all subjects and they were interviewed regarding age, medical history, possible chemical exposure, acute or chronic diseases, smoking and dietary habits.

*Blood sampling*

Briefly, 4 mL of venous blood samples were collected in K_3_EDTA-containing tubes (Vacutainer® Becton Dickinson). The subjects were asked for blood sampling at the same time about 10 am and samples were stored at −20 °C until further analysis.

*Instrument*

The blood cadmium and lead concentration were measured by double beam GBC plus 932 atomic absorption spectrometer (GBC Scientific Equipment Pty Ltd., Australia) equipped with pyrolytic coated graphite tubes, autosampler PAL 3000 and deuterium background effect correction. In all determinations steps, argon of 99.998% purity (200 mL/min) was used as a sheet gas except during the atomization step in which the purge gas flow was interrupted. Throughout the procedure absorbance values of both peak height and peak area were measured. The blood sample was mixed with acidic solution in the Teflon vessels of Anton PaarMultiwave 3000 (Graz, Austria) microwave oven and digested by the heating program. The GBC Avanta software version 2.02 was used for calculation and statistical evaluation.

*Reagents and solutions*

All reagents used were of analytical grade and purchased from Merck (Darmstadt, Germany) unless otherwise mentioned. All glassware and plastic instrument were completely immersed for 24 h in 2 M nitric acid then followed by washing with deionized water. Ultrapure de-ionized water obtained from a MilliQwater purification system (Millipore, Bedford, USA) was used throughout the experiment. Concentrated Nitric acid 65%, Hydrogen peroxide 30%, Hydrochloric acid 37% were used for sample preparation and acid digestion. Stock solutions of Pb and Cd with a concentration of 1 g L^−1 ^(Merck Millipore, Darmstadt, Germany) were used to prepare 100 mg L^−1^ standard solution during the measurement. Seronorm^TM^ Trace Elements Whole Blood Level 1 (210105), Whole Blood Level 2 (210205), Whole Blood Level 3 (210305) from Sero AS (Billingstad, Norway) as internal quality controls were used to ensure the accuracy of measurement.

*Sample preparation and analytical procedure*

For GFAA analyses, the samples matrix was destroyed by microwave digestion. For this purpose mixture of blood, HNO3 and H2O2 were transferred into a pressure resistant PTFE vessel (For this purpose 1 mL of blood samples, 3 mL of concentrated HNO_3_ and 1 mL of concentrated H2O2 were transferred into pressure resistant PTFE vessel). The samples were then digested following three steps program: (i) 20 min at 150 ºC and 50 % power; (ii) 40 min at 220 ºC and 70% power and (iii) 15 min at 100 ºC and 10% power. The resulting colorless mixture was transferred to the volumetric flasks and diluted with deionized water. The commercially purchased quality control solutions (Certified Reference Materials known as CRM) were decomposed using the same digestion program as stated above. 

The instrumental parameter and operating condition were performed for measuring the elements throughout this work are presented in Tables 1. and 2. Five aqueous standards were used to obtain the calibration curves. The total volume of 20 µL (10 µL of sample solution and 10 µL of the modifier solution) was injected into the tubes by means of autosampler. As a rule, three replicates (n = 3) were measured for each sample.

*Statistical analysis*

Frequency distribution and chi-square test were used to summarize qualitative variables. Student’s t-test was performed to compare group means. Kruskal-Wallis and Mann-Whitney as Nonparametric tests were then used to compare heavy metals concentration across measured variables. All statistical evaluation and data processing were carried out by using GraphPad Prism software (La Jolla, CA, USA). Results are expressed as means ± SD. Values of p˂0.05 were defined as statistically significant.

## Results

The study population comprised 66% female and 34% male subjects in which, the mean age of them were 34.8 ± 10.7 and 31.5 ± 11.1 years, respectively. Moreover, the proportions of smoker subjects were 22.4% in the total study population. Study participant’s characteristics classified by sex, age and smoking habits in each group are shown in Table 3.

*Blood lead and cadmium level*

A significant difference was observed in cadmium level (Mann-Whitney test; p = 0.041) in which, MS patients had higher blood concentration in comparison with healthy individuals (1.8 ± 0.13 μg/L vs. 1.4 ± 0.11 μg/L). With respect to Lead, there was no significant difference between cases and controls (p = 0.625). The summarized descriptive statistics of blood lead and cadmium concentrations in the study population are shown in Table 4.

**Figure 1:**
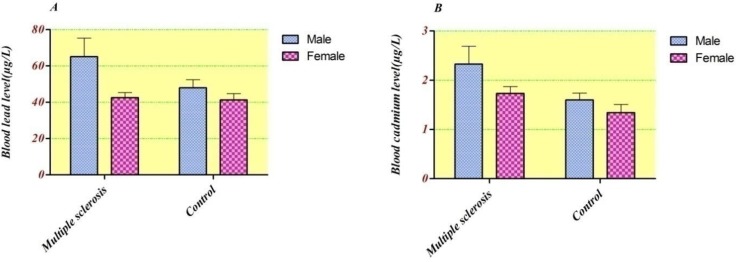
Blood concentration of A) Lead (µg/L), B) Cadmium (µg/L) compared between groups based on gender ratio.

The blood lead and cadmium concentrations in accordance with group’s gender distribution were checked (Figure 1.). It was found that in all subjects males had higher blood metals level. The geometric means of blood lead concentration were considerably higher in males than in females in MS patients (57.1 ± 33.7 μg/L vs. 36.7 ± 21.9 μg/L. P = 0.02). However, there were no considerable differences in cadmium level between males and females in both groups (ANOVA: F = 2.66 and F = 0.69; p˃0.05, respectively).

**Figure 2:**
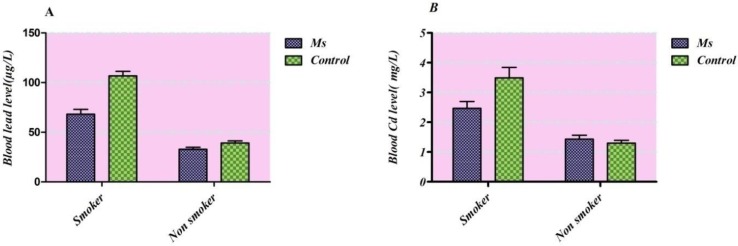
Frequency distributions of the blood lead (a) and cadmium (b) levels (μg/L) taking smoking consideration in each group

**Table 1:** Instrumental settings applied for determination of Pb and Cd by ETAAS.

**Analyte**	**Calibration mode**	**Wavelength (nm)**	**Slit width (nm)**	**Lamp current (mA)**
Lead	Standard curve	283.2	1	5
Cadmium	Standard curve	228.8	0.5	3

**Table 2:** Optimized graphite furnace heating parameters for Pb and Cd assessment in the blood sample by using Mg (NO3)2 as a chemical modifier

**Analyte **	**Step **	**Temperature (ºC) **	**Ramp(s) **	**Holds(s) **	**Gas flow rate ** **(L/min) **
Lead	Preheating	110	5	10	3
	Drying	130	5	10	3
	Pyrolysis	700	10	15	3
	Atomization	1800	0	4	0
	Cleaning	2400	2	2	3
	Cooling	40	25	5	3
Cadmium	Preheating	110	1	10	3
	Drying	130	5	20	3
	Pyrolysis	700	10	15	3
	Atomization	1400	0	5	0
	Cleaning	2200	1	2	3
	Cooling	40	25	5	3

**Table 3:** Characteristics of the study population

**Groups**	**Parameter**	**Male**	**Female**	**Total**
Multiple sclerosis patients	No of participants	11 (15.9%)	58 (84.1%)	69
	Age (years)*	33.2±12.7	35.5±10.6	35.2±10.9
	Current smoker	8 (30.7%)	18 (69.2%)	26
	Non smoker	3 (6.9%)	40 (93.0%)	43
	Disease duration	7.3±6.1	7.4±4.8	7.4 ± 4.9
	(years)	(1-21)	(1-23)	(range 1-23)
Healthy individuals	Disease severity	3.5±1.9	1.9±1.2	2.2 ± 1.43
	(EDSS)	(0.5-6.5)	(0.5-6.0)	(range 0.5-6.5)
	No of participants	38(52.0%)	36(48.0%)	74
	Age (years)	29.7±9.4	33.8±10.8	31.8±10.3
	Current smoker	4 (66.6%)	2 (33.3%)	6
	Non smoker	34 (50.0%)	34 (50.0%)	68

* Values are expressed as mean ± SD

**Table 4:** Blood lead and cadmium level in the study population

**Groups name **	**Lead **					**Cadmium **					
	Mean	Min	Max	SEM	Median	Mean		Min	Max	SEM	Median
Control	44.7	15.6	124.8	2.9	35.4	1.47	a		4.7	0.1	1.3
MS	46.1	11.2	123.8	3.1	43.5	1.8	a		5.6	0.1	1.6
P value	0.625					0.041*					

a under limit of detection.

However, there was no significant difference in lead level between cases and controls and cadmium between males and females in both groups. Taking into consideration tobacco smoking habits of study participants in both sexes together (Figure 2.), an elevated Cd and Pb blood levels were observed in smokers in comparison with non-smokers (p<0.0001 for both groups).

## Discussion

The sharp increase in MS prevalence rate was reported in Iran (11) and it seems that Iran’s prevalence rate is similar to some European countries (17). Tehran is the capital of Iran with an estimated population of 14 million where, rapid industrial and economical developments have been resulted in an increase of environmental pollution (1). A joint point trend analysis by Heydarpour *et al.* showed a statistically significant increasing trend with Annual percent change (APC) of 8.39% in both genders (95% CI: 6.6-10.2%) (19). In addition, the incidence rate of MS in Tehran is going up from 0.68 per 100000 people in 1989 to 4.58 per 100000 in 2005 (19). It has been suggested that focal plaque-like demyelination in multiple sclerosis is related to excessive oxidative damage, but the main cause of oxidative injury in MS is still unknown. However, increased sensibility of neuronal process and oligodendrocytes through the elevated inflammatory process could be related to oxidative stress (20). On the other hand, it has been proved that toxic metals by producing harmful free radicals participating in protein and DNA modification, lipid peroxidation and breakdown of blood brain barrier (5).

In case of multiple sclerosis patients, we found that the blood cadmium concentration was significantly higher in comparison with healthy individuals (1.82 ± 0.12 vs 1.47 ± 0.11, p = 0.006). Recent ecological risk assessment which was done in Tehran reflected an alarming street dusts containing cadmium pollution (21). Various source of exposure to cadmium as redox inert element with unknown biological effect in drinking water, air and food in contaminated areas. it has been suggested that Cd toxicity leads to severe organs damage such as a brain, testis, kidney, lung and liver (9). In addition, Cd induces neurological abnormalities, neonatal cerebral edema and cerebral hemorrhage in animal experimental studies (10). Moreover, Cd has been reported to increase the production of reactive radicals and interferes with antioxidant enzymes activity in adult rat brain. This effect in turn results in alteration of membrane-bound enzymes including Na+/K+ ATPase and structural lipids integrity (22, 23). In developing rat, it has been observed that initially Cd changes the vascular endothelium permeability resulting in focal edema, brain oxygen and nutrient uptake interference and finally the necrotic changes in neuronal components which are secondary to this effect (10, 24, 25).

According to Table 4. there was no significant difference between cases and controls in lead blood concentration (46.1 ± 3.0 and 44.6 ± 2.8 respectively, p = 0.62). Environmental lead pollution has risen rapidly due to using million tons of leaded gasoline especially in industrial areas (6). Lead acts as redox active metals and generates free radical species by taking a part in electron transfer, while redox inert metals such as Cd mainly depletes the reduced glutathione and binding to thiol group of various proteins (5, 26). Lead has a multifactorial pathogenic role including direct generation of various ROS such as hydrogen peroxides, singlet oxygen and hydroperoxides and deplete the cellular antioxidant sulphydryl pool (8). 

As shown in Figure 1. considering the whole blood level, each metal were slightly higher in all men subjects than women, however, the GM of lead blood concentration in male subjects showed statistically significant increase in comparison with females (51.8 ± 4.3 vs. 42.0 ± 2.2, p = 0.026). However, there were no statistically significant differences between Cdblood concentrations of males and females in both groups (ANOVA: F = 2.66 and F = 0.69; p˃0.05, respectively). In line with our results, some of other studies suggested such differences (27-29). Entirely, men because of the higher amount of RBC, higher exposure and gender-related differences in lead metabolism have elevated blood lead concentration than women (30). Our results are in agreement with recent research in Tehran population, in which the total blood concentrations of Cd and Pb in males are higher than in females. In addition, the obtained mean blood cadmium level in our study (1.78 µg/L) is comparable to this study (1.82 µg/L) (1).

Overall the main sources of Cd and Pb exposure in Tehran residents are air-suspended particles (31). In which it may worsen in winter because of local topography and high industrial activities. It has been observed that prolong exposure to air pollutants in Tehran disclosed as an environmental risk factor in MS prevalence (32). It has been proved that water contamination and rice heavy metal content also causes an increase in cadmium body burden (33-35). On the other hand, food and tobacco smoking are the main sources of cadmium exposure in the absence of occupational situations (36, 37). Taking into consideration tobacco smoking habits of study participants in both sexes together in Figure 2. an elevated contents of cadmium and lead were observed in blood specimen of smokers in comparison with non-smokers (p<0.0001 for both groups). It was reported that smoking affected lead body burden thorough changing bone deposition while assessed direct cadmium level was roughly two-fold higher in smokers subjects (38-40).

Nowadays cigarette smoking is one of the modern habits across the world especially in developing countries though there is an alarming increase in incidence rate of smoking between younger women (41). According to recent studies, women have the higher risk of developing MS and the gender ratio has changed considerably in Iran over the last years (42). There is some research suggested that tobacco smoking alters the immune system and blood leukocyte counts which mean it may affect inflammatory markers (43, 44). In addition, some studies points to blood brain barrier alteration associated to nitric oxide free radical production regarding cigarette smoking (45). A significant correlation between cigarette smoking and risk of multiple sclerosis has been shown (46, 47). But the exact mechanism of cigarette and its correlation with MS development is still unknown. Based on our results it is reasonable to infer that heavy metals especially lead and cadmium is associated with the toxicity of tobacco products as well as MS development. 

In conclusion, on the basis of our results, blood cadmium level was higher in multiple sclerosis patients in comparison with healthy individuals. Manouchehrinia *et al*. suggested the possible relation between premature mortality and tobacco smoking in MS patients(48). On the other hand, in this study it was shown that there is a significantly elevated cadmium level in patients’ blood sample. It could be inferred that various cadmium exposure might be affected susceptibility to multiple sclerosis and could increase its risk of development.
